# Case report: Complex arterial findings in vascular ehlers-danlos syndrome with a novel *COL3A1* variant and death at young age

**DOI:** 10.3389/fcvm.2023.1110392

**Published:** 2023-06-19

**Authors:** Jacopo Taurino, Emanuele Micaglio, Annalisa Russo Raucci, Monica Zanussi, Massimo Chessa, Nathasha Samali Udugampolage, Paola Carrera, Carlo Pappone, Alessandro Pini

**Affiliations:** ^1^ Cardiovascular-Genetic Center, IRCCS Policlinico San Donato, Milan, Italy; ^2^ Arrhythmology & Electrophysiology, IRCCS Policlinico San Donato, Milan, Italy; ^3^ Laboratory of Clinical Genomics, IRCCS San Raffaele Scientific Institute, Milan, Italy; ^4^ ACHD Unit - Pediatric and Adult Congenital Heart Centre, IRCCS-Policlinico San Donato, Milan, Italy; ^5^ Vita & Salute San Raffaele University, Milan, Italy; ^6^ Genomics for the Diagnosis of Human Pathologies, IRCCS San Raffaele Scientific Institute, Milan, Italy

**Keywords:** *COL3A1*, vascular ehlers danlos syndrome, aneurysm - dissecting, pneumothorax, atypical onset

## Abstract

Vascular Ehlers-Danlos syndrome (vEDS) is a genetic disease caused by a pathogenic mutation in the *COL3A1* gene. Despite its severe course, the rarity and extreme clinical variability of the disease can pose significant obstacles to a timely diagnosis. Early and accurate diagnosis may lead to improved patient outcomes by providing access to targeted pharmacological treatments like celiprolol and enhancing the management of vEDS-related complications. Herein, we report a patient harboring a novel *de novo COL3A1* missense variant, in which the diagnosis was only possible belatedly due to delayed referral for genetic evaluation. The patient developed pulmonary complications, aneurysms, and vascular malformations, and died at the age of 26 years due to massive pulmonary bleeding.

## Introduction

Vascular Ehlers-Danlos syndrome (vEDS) is a rare connective tissue disorder characterized by a severe course involving fragility and easy rupture of arteries and bowel, transparent skin, easy bruising, and distinct facial features such as protruding eyes, thin nose and lips, sunken cheeks, and lobeless ears (1).

Respiratory manifestations of vEDS are uncommon but have been described, including spontaneous pneumothorax, intrapulmonary bleeding causing recurrent hemoptysis, and bulla or bleb formation (2).

vEDS can be life-threatening at a very young age, and its diagnosis is challenging, particularly in the absence of a family history, due to the rarity and extreme clinical variability associated with the disorder (3). However, primary diagnosis of vEDS can be suspected through careful genetic counseling based on clinical presentation and then confirmed by identifying the pathogenic variant in the *COL3A1* gene.

## Case report

The patient was born preterm at the 35th week of gestation due to premature rupture of membranes. At just four months of age, he experienced an apparent life-threatening event (ALTE) while crying, characterized by sudden loss of consciousness, generalized hypotonia, and cyanosis (no documentation was available). Two months later, a similar event occurred, and a head CT scan revealed bilateral subdural hematoma that was resolved via craniotomy. As a result, the patient suffered reduced visual acuity in the left eye, believed to be secondary to compression of the ipsilateral optic nerve, but he did not develop any other permanent neurological deficits over time.

Starting at the age of 14 years, the patient began experiencing recurrent episodes of hemoptysis, accompanied by right-sided paresthesias, headache, and photophobia. A brain MRI revealed cerebellar tonsil ectasia through the foramen magnum. That same year, the patient suffered a spontaneous right-sided pneumothorax, which was treated with mechanical pleurodesis and exeresis of a bullous lesion. Another pneumothorax occurred at the age of 17 years, this time managed with chemical pleurodesis. Between these two events, he underwent saphenectomy to address multiple vein ectasia in his left leg.

At 22 years old, after an episode of severe hemoptysis, an exploratory thoracotomy was performed, revealing a massive spontaneous right-sided hemothorax. Following suspicion of a coagulation disorder, a hematologic screening which included CBC, PT, aPTT, PFA-100, coagulation factor assay, and vWF dosage—was carried out; all results were normal. At the time of testing, vEDS was not suspected and genetic counseling was not advised. Lacking a definite diagnosis, the patient continued his active lifestyle without adopting any cautious behavior, including smoking.

At the age of 25 years, the patient presented to the emergency department of our institute with acute pain in the right thoraco-abdominal area. A thoracic-abdominal CT scan revealed a massive retroperitoneal hematoma in the right perirenal region, extending from the subdiaphragmatic region to the pelvic cavity (maximum diameter of 30 cm), adjacent to a large anastomotic circle between the aorta and the right pulmonary artery. Multiple dilations of the peripheral branches of the pulmonary artery were also observed.

**Figure 1:**
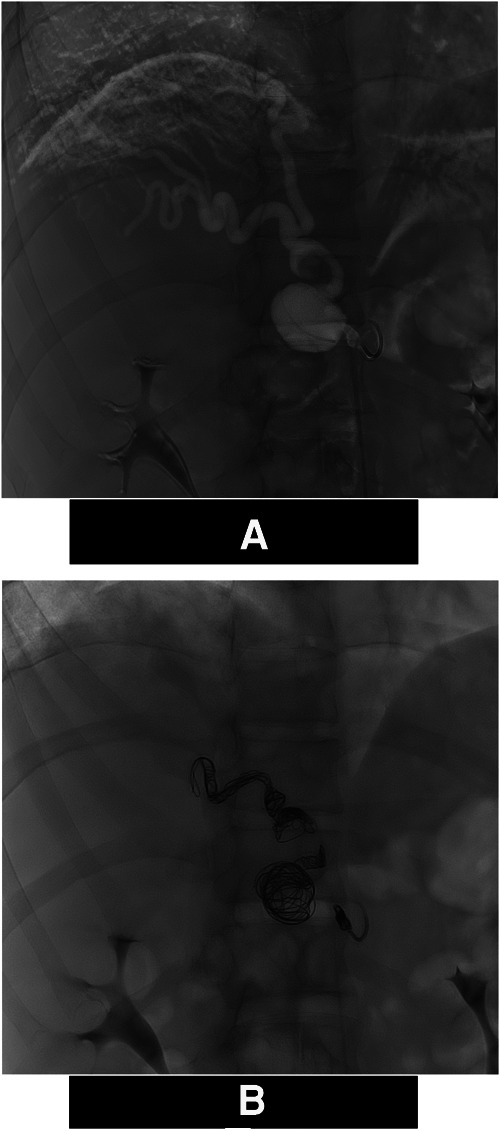
Angiograms of the right inferior diaphragmatic artery. (**A**) Digital Subtraction Angiography Hypertrophy of the right inferior diaphragmatic artery and evidence of 2 aneurisms (*). (**B**) Angiogram: Post Coil embolization using Interlock mechanically detachable coils (Boston Scientific, Natick, MA) and The Concerto™ Helix and 3D detachable coil systems (Medtronic Inc Minneapolis MN, US).

**Figure 2:**
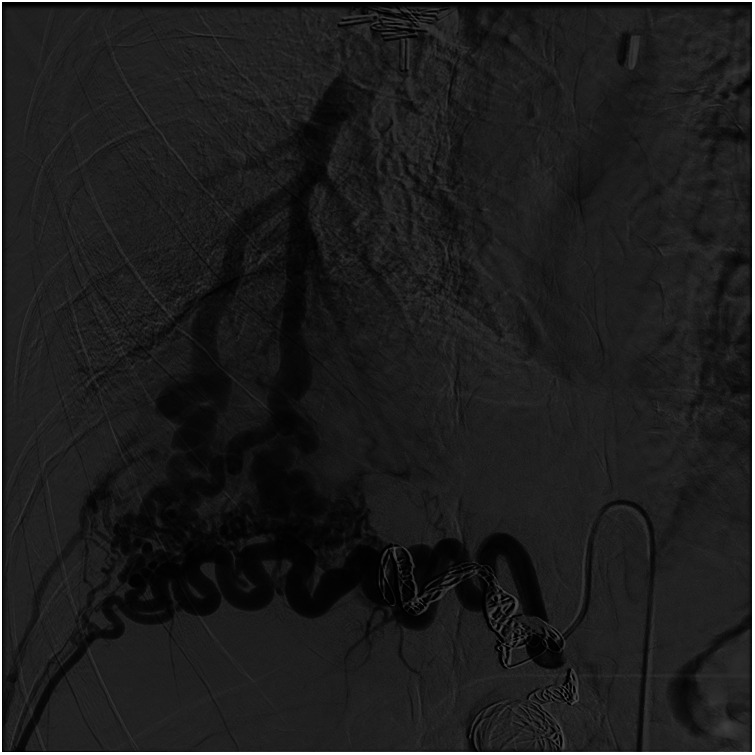
Digital subtraction angiography. Intercostal hypertrophic artery (*) and artero, venous fistula between this artery and the inferior right pulmonary vein (#).

The right diaphragmatic artery appeared hypertrophic, circumvoluted, with two aneurysmal formations one of which was also partially thrombosed. These findings were confirmed by the angiograms (Figure 1A) and treated via selective coil embolization (Figure 1B). The angiograms also revealed a small dissection flap in the right external iliac artery, and a hypertrophic and circumvoluted 12th intercostal artery. Later, selective catheterization of this artery demonstrated arterial-venous shunts between it and the pulmonary vein for the right lower pulmonary lobe (Figure 2). The shunts were then treated via selective embolization as well (Figure 1B).

One month later, the patient was readmitted to the emergency department due to another episode of hemoptysis. The chest CT scan revealed “ground glass” consolidation areas with a central consolidated component in the lung parenchyma, with the largest located at the left posterior costophrenic sinus, and several smaller ones in the left lower pulmonary lobe.

These findings were consistent with alveolar hemorrhage, likely originating from the hypertrophic left bronchial artery, where a pseudoaneurysm was recognizable. A subsequent angiography showed markedly tortuous and ectasic proximal sections of the bronchial arteries and hyperemia of the distal bronchial circles.

Superselective catheterization attempts to reach the bleeding site of the left bronchial artery were unsuccessful. Consequently, as the hemoptysis resolved, the patient was not subjected to an embolization procedure; instead, he was started on tranexamic acid therapy without a clinical indication for whole blood transfusion.

During this hospitalization, the patient was referred to clinical genetics for further evaluation. Upon physical examination, the patient exhibited a muscular constitution; his height was 177 cm, and his weight was 75 kg. The skin assessment was challenging due to the presence of multiple tattoos covering the trunk and limbs. No facial anomalies or signs of joint hypermobility were evident; however, a high-arched palate, absence of the lingual frenulum, and flat feet with hammer toes were observed. A connective tissue disorder was finally suspected.

**Figure 3:**
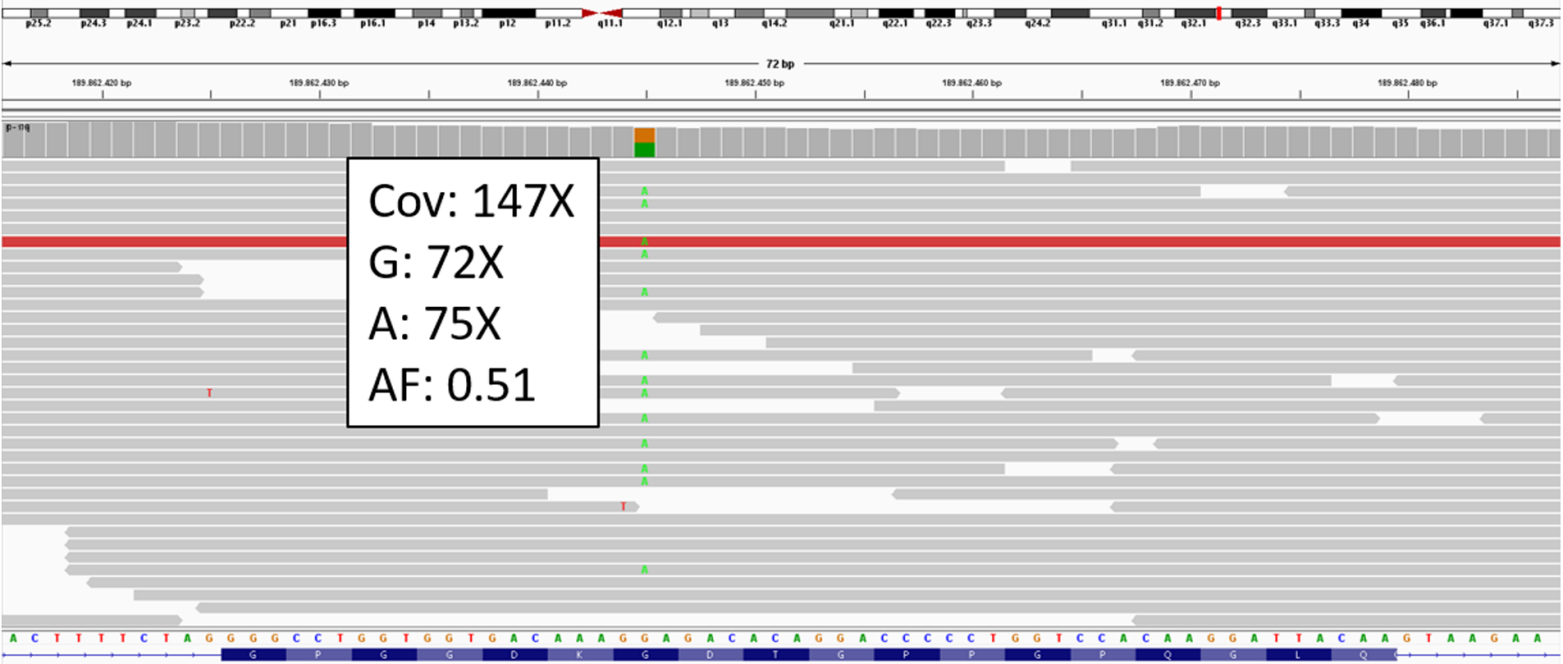
*COL3A1* BAM from IGV. The c.1889G > A variant is detected in heterozygosity. Cov: coverage; AF: allelic fraction of alternative called base (**A**).

To verify this hypothesis, the patient underwent genetic testing from peripheral blood extracted DNA using a panel of 20 genes (*ACTA2, COL1A1, COL1A2, COL3A1, COL5A1, COL5A2, ELN, FBN1, FBN2, FLNA, MYH11, MYLK, NOTCH1, PLOD1, SMAD3, TGFB1, TGFB2, TGFBR1, TGFBR2, TNXB*) via next-generation sequencing. The analysis identified the novel heterozygous missense variant c.1889G > A causing the amino-acid substitution p.(Gly630Glu) in *COL3A1* (NM_000090.3) (Figure 3). Subsequent testing of parental samples demonstrated that the variant originated *de novo* (Supplementary Figure S1). A literature search (PubMed, Human Gene Mutation Database, LOVD, Ehlers-Danlos Syndrome variant Database, Internal data, ClinVar) confirmed that this variant has not been previously reported, indicating a novel mutation.

According to the ACMG standards for variant classification (4), the following criteria were applied to classify the variant as ‘likely pathogenic’: the variant was absent in both the unaffected parents (PS2), it is absent in the gnomAD database (PM2); it is located within the collagen triple helix repeat (PM1), and it is predicted in silico as deleterious by many different tools, including MetaRNN, M-CAP, MVP, Polyphen2 HDIV, Polyphen2 HVAR, SIFT, DEOGEN2, EIGEN, FATHMM, and MutPred (PP3).

The patient’s clinical presentation, along with imaging and genetic test results, led to a diagnosis of vEDS, and pharmacological therapy with celiprolol 200 mg daily was started.

The patient was subsequently evaluated by a multidisciplinary team to determine the best approach for treating the frequent pulmonary hemorrhages.

The only resolutive treatment proposed was heart-lung transplantation. However, the surgical team deemed this option impossible due to the patient's previous chemical pleurodesis and tissue fragility. Later that year, the patient experienced a new spontaneous pneumothorax, followed by a massive pulmonary hemorrhage that proved fatal at the age of 26 years.

## Discussion

Vascular Ehlers-Danlos syndrome (vEDS) is the most severe subtype among the Ehlers-Danlos syndromes (5); caused by pathogenic variants in the *COL3A1* gene, which encodes the *α*1 chain of type III procollagen [pro*α*1(III)].

vEDS carries a high risk of sudden death due to the rupture of large arteries or hollow organs, such as the bowel and uterus. The median age for the first major vascular event is 24.6 years, and the median survival is only 48 years due to the high rate of complications (6).

Clinical diagnostic criteria for vEDS include: a positive family history of the disorder, early onset of arterial rupture or dissection before age of 40 years, unexplained rupture of the sigmoid colon, unexplained spontaneous pneumothorax or carotid-cavernous sinus fistula formation without trauma, and minor vEDS features (e.g., tendon and muscle rupture, easy bruising, thin translucent skin, hypermobility of small joints, aged appearance of extremities particularly the hands, gingival recession, keratoconus, early onset varicose veins) (5).

Imaging, including computed tomography angiography and magnetic resonance angiography, is an essential tool for diagnosing and monitoring vEDS. Aneurysms, dissections, arterial ectasia, and occlusions are the most common radiologic findings, with abdominal vessels being the most frequently affected (34%), followed by thoracic (33%), and head/cervical vessels (17%) (6).

Genetic testing is the definitive method for confirming the diagnosis of vEDS, through the identification and characterization of a *COL3A1* pathogenic variant, which may also offer a prognostic insight. According to Omar et al. (7), the majority of pathogenic variants in the *COL3A1* gene (approximately 65%) are missense variants, resulting in the substitution of a glycine residue within the triple helical domain. Aberrant splicing, occurring in about 25% of patients, is the second most common mutation mechanism. Additionally, 5% of patients have nonsense or frameshift variants, leading to unstable RNA that is degraded through nonsense-mediated decay without producing protein (null mutations or haploinsufficiency).

According to Pepin et al. (6), splicing variants are associated with the most severe disease course, followed by missense variants involving glycine residues, and finally haploinsufficient variants are associated with a milder course, including delayed symptom onset and increased life expectancy.

Diagnosing vEDS can be challenging, especially because 50% of *COL3A1* pathogenic variants occur *de novo*. As a result, vEDS is often only suspected after a significant or potentially fatal complication occurs (3).

For 70% of vEDS patients, the presenting sign is either a vascular rupture or an organ rupture (8). However, this means that a significant portion of patients have an atypical presentation of vEDS. Among these, 12% of patients have a spontaneous pneumothorax due to the rupture of a lung bleb as their first described manifestation (3). Additionally, pulmonary hemorrhages related to the rupture of small vascular nodules or arteriovenous fistulas have been reported as well (9, 2).

In 2015, Abrahamsen et al. (10) described a patient carrying a *COL3A1* pathogenic variant whose disease onset occurred at the age of 19 years, characterized by recurrent episodes of spontaneous pneumothorax. The patient also had other typical signs of the disease, such as a tall and slender build, mild scoliosis, asymmetry of the chest, long fingers with hypermobility of the thumb joints, thin and translucent skin, prominent eyes, and keratoconus, which further supported genetic testing for *COL3A1*. The authors suggested that the atypical presentation of vEDS with pneumothorax and pulmonary bleeding may be more common in young adults.

Treatment for vEDS primarily focuses on avoiding high-risk activities and procedures such as arterial punctures, elective surgeries, and gastrointestinal and uterine endoscopies. These procedures should be limited to cases with urgent complications, including arterial or organ rupture. Blood pressure control is crucial, and the use of antiplatelet and anticoagulation medications should be avoided unless strictly indicated. It is essential for patients to understand their risk factors and implement necessary lifestyle changes to reduce the risk of complications, such as avoiding strenuous physical activity and contact sports (8).

Atypical presentations of vEDS may delay diagnosis, resulting in suboptimal patient management and serious clinical consequences. In this regard, Diebels et al. (11) described a 58-year-old patient with Stanford type B aortic dissection who underwent thoracic endovascular repair (TEVAR) but experienced a retrograde dissection with rupture of the ascending aorta just two days later, which ultimately proved fatal. In this case, diagnosis was not possible through clinical criteria, but was only revealed post-mortem thanks to the identification of a pathogenic mutation in the *COL3A1* gene. The authors emphasized the importance of a conservative management approach for vascular events in patients even suspected of having vEDS, given the high fragility of their tissues and blood vessels, which can lead to very serious complications.

Currently, the only medical therapy that has been shown to improve outcomes for vEDS is the use of celiprolol, a β1-adrenoceptor antagonist and a β2-adrenoceptor agonist that reduces hemodynamic stress and the fragility of arterial walls (12).

The extent of evaluation at the time of initial diagnosis is currently a topic of ongoing debate. The most important step in managing vEDS is assembling an interdisciplinary care team, which should include a primary care physician, a geneticist, a vascular surgeon, and a general surgeon. This team will be responsible for coordinating both routine and emergency care (8).

In this work, we describe a patient with vEDS who died from the disease at a young age. Recurrent spontaneous pneumothorax and pulmonary hemorrhage in young individuals may indicate an atypical onset of vEDS and should warrant prompt genetic counseling, even in the absence of other common symptoms such as facial, skin, and joint anomalies.

Unfortunately, genetic counseling for our patient was recommended only after his condition worsened, and the delayed diagnosis precluded the proper clinical management that might have been potentially beneficial.

An earlier diagnosis might have allowed for timely treatment with celiprolol, which has been associated with better cardiovascular outcomes and longer life expectancy in vEDS patients. Furthermore, it might have led to alternative management strategies for the patient's recurrent pneumothoraces.

In this scenario, the typical tissue fragility of vEDS alone was not the reason to contraindicate heart and lung transplantation in our patient. However, this fragility, combined with the result of the previous chemical pleurodesis led the surgical team to deem any intervention unfeasible.

## Conclusion

The usefulness of this case is to demonstrate the pivotal importance of referring to clinical genetics evaluation for all patients with a history of recurrent bleedings and spontaneous pneumothoraces. Early diagnosis of vEDS may enable better treatment choices, improved management, and ultimately, better outcomes.

## Data Availability

The original contributions presented in the study are included in the article/Supplementary Material, further inquiries can be directed to the corresponding author.
