# Supplementary material for: Case report: Complex arterial findings in vascular ehlers-danlos syndrome with a novel COL3A1 variant and death at young age
Source: Front Cardiovasc Med. 2023 Jun 19;10:1110392. doi: 10.3389/fcvm.2023.1110392 (PMC10315819; doi:10.3389/fcvm.2023.1110392)
Supplement: Supplementary file 1 [file Table1.docx]

**Table S1.** Summary of Proband's Clinical History.

| **Age** | **Event** |  |
| --- | --- | --- |
| 0 | Born preterm at 35th gestational weeks for premature rupture of membranes |  |
| 4 months | Apparent life-threatening event (ALTE) |  |
| 6 months | Bilateral subdural hematoma requiring craniotomy |  |
| 14 years | Spontaneous right-sided pneumothorax |  |
| 14-22 years | Recurring episodes of hemoftoe usually preceded by right hemilateral paresthesias, headache and photophobia |  |
| 15 years | brain MRI that showed cerebellar tonsil ectasia |  |
|  | saphenectomy due to multiple vein ectasia in left leg |  |
| 17 years | Second pneumothorax treated with chemical pleurodesis |  |
| 22 years | | Exploratory thoracotomy due to massive hemoptysis  Negative ematologic screening |
| 25 years | | Massive retroperitoneal hematoma. |
|  |  | Identification of   - multiple dilations of the peripheral branches of the pulmonary artery - two aneurysmal formations of the right diaphragmatic artery - small dissection flap at the level of the right external iliac artery - hypertrophic and circumvoluted appearance of the right diaphragmatic artery and of the 12th intercostal artery.   arterial-venous shunts with opacification of the pulmonary vein for the right lower pulmonary lobe |
|  |  | Selective embolization of the right diaphragmatic artery and of the last right intercostal artery |
|  |  | Alveolar hemorrhage -> started tranexamic acid |
|  |  | Referral to clinical genetics -> Identification of COL3A1 de novo mutation -> Diagnosis of vEDS |
| 26 years | | Third Spontaneous Pneumothorax |
|  |  | Massive pulmonary hemorrhage and death |
