# Supplementary material for: Case report: Complex arterial findings in vascular ehlers-danlos syndrome with a novel COL3A1 variant and death at young age
Source: Front Cardiovasc Med. 2023 Jun 19;10:1110392. doi: 10.3389/fcvm.2023.1110392 (PMC10315819; doi:10.3389/fcvm.2023.1110392)
Supplement: Supplementary file 2 [file Datasheet1.pdf]

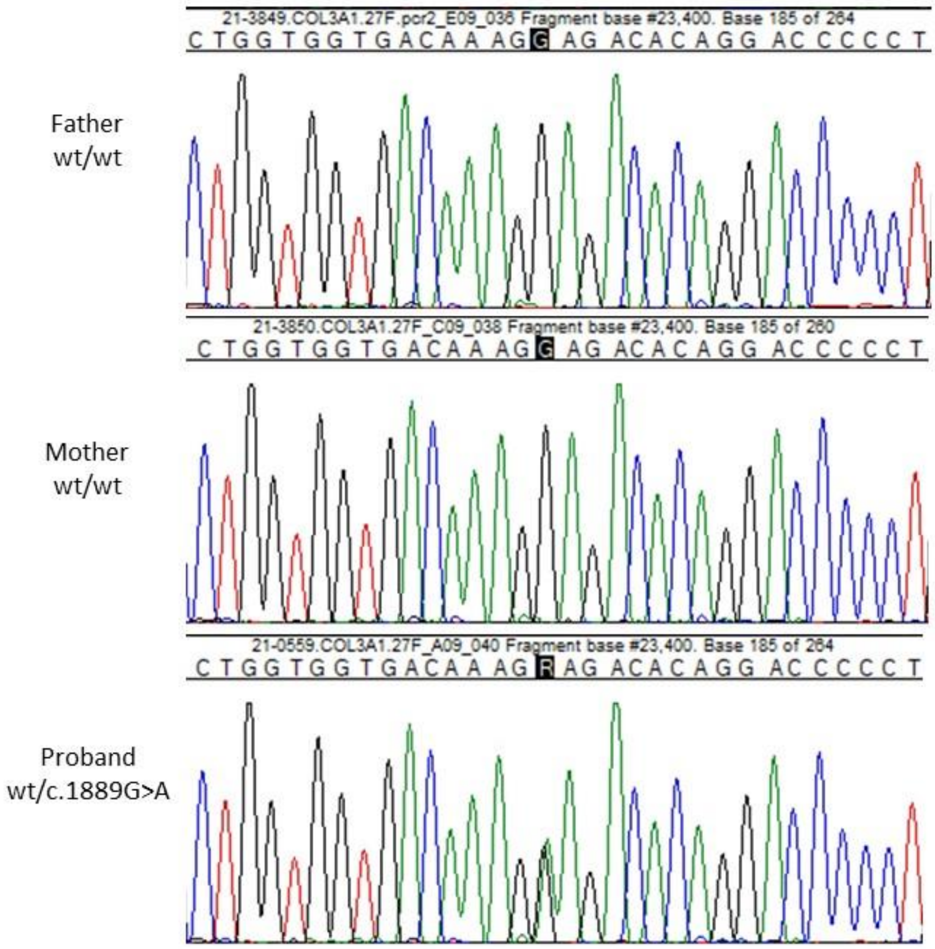

Figure S1. Electropherograms of the Parents and Proband. The electropherograms show the presence of the *COL3A1* variant in the proband and its absence in both parents.
